# Fabrication of a low-adhesion metallic cell culture surface by nanosecond laser processing

**DOI:** 10.1007/s00449-025-03268-5

**Published:** 2025-12-20

**Authors:** Kaisei Ito, Atsushi Ezura, Hideharu Shimozawa, Yoshikatsu Akiyama, Chikahiro Imashiro, Jun Komotori

**Affiliations:** 1 School of Science for Open and Environmental Systems, Graduate School of Science and Technology, Keio University, 3-14-1 Hiyoshi, Kohoku-ku, Yokohama, Kanagawa 223-0061 Japan; 2 Sanjo City University, 5002-5 Kamisugoro, Sanjo-shi, Niigata 955-0091 Japan; 3 Medical Systems and Components Operations, Canon Inc., 3-30-2 Shimomaruko, Ota-ku, Tokyo, 146-8501 Japan; 4 Institute of Advanced Biomedical Engineering and Science, Tokyo Women’s Medical University (TWIns), 8-1 Kawada-cho, Shinjuku-ku, Tokyo, 162-8886 Japan; 5 Graduate School of Engineering, The University of Tokyo, Hongo 7-3-1, Bunkyo-ku, Tokyo, 113-8654 Japan; 6 Department of Mechanical Engineering, Keio University, 3-14-1 Hiyoshi, Kohoku-ku, Yokohama, Kanagawa 223-0061 Japan

**Keywords:** Cell production, Enzyme-free cell detachment, Low-adhesion culture surface, Reusable metallic culture surface, Cell sheet technology, Nanosecond laser processing, Microtopography surface, Surface Texturing

## Abstract

In this study, we developed a reusable low-adhesion metallic cell culture surface having microscale structures using nanosecond pulsed laser processing. Titanium alloy disks were mirror-polished and laser-processed to create microstructures with a pitch of 15 μm, smaller than typical cell size. The cytocompatibility of the developed surfaces was confirmed, showing comparable viability to standard plastic dishes. On the other hand, the cells on the laser-processed surfaces exhibited suppressed lamellipodia formation and maintained a rounded morphology and the area of adhered cells was significantly inhibited compared to polished surfaces, indicating reduced adhesion. Further, by applying PBS jet flow to the culture surface, it has been demonstrated that the cells on the micro-structured surfaces formed significantly larger detachment zones under PBS jet flow, confirming weakened adhesion strength. Furthermore, intact cell sheets could be detached from the laser-processed surfaces by pipetting, whereas cells on polished surfaces remained adherent. These results suggest that the developed culture surface enables on-demand cell detachment through physical stimuli without enzymatic treatment, maintaining cell–cell junctions and extracellular matrix integrity. This technology offers potential for applications in cell sheet engineering and enzyme-free cell harvesting, contributing to cost-effective and sustainable cell-based applications. Future work should investigate cell proliferation and migration behavior to further validate its utility for industrial tissue engineering platforms.

**Supplementary Information:**

The online version contains supplementary material available at 10.1007/s00449-025-03268-5.

## Introduction

In recent years, numerous studies have been conducted toward the practical implementation of cell-based applications. Among these, one of the most critical and universal processes is the detachment of adherent cultured cells. Conventionally, enzymatic detachment methods using proteolytic enzymes such as trypsin are employed; however, these methods inevitably damage the cell membrane and adhesion molecules, potentially compromising cell viability and functionality [1–6]. As a result, enzyme-free detachment techniques utilizing temperature-responsive polymers [7, 8] or ultrasound [9, 10] have been actively developed in recent years. However, for certain cell species such as strongly adherent cells, these enzyme-free methods are often ineffective. Therefore, strategies that moderately suppress adhesion to the cell culture surface—while still maintaining sufficient adhesion to enable cell culture—should be investigated. One approach involves the permanent inhibition of cell adhesion using chemical modifications such as PEG grafting [11, 12] or pHEMA coating [13, 14]. However, these methods block protein adsorption entirely, which often prevents normal cell adhesion and proliferation, making them unsuitable for cell culture.

To overcome this problem, methods that reduce adhesion area by fabricating microstructures smaller than the size of cells—such as pillar-like topographies—on the culture surface have been proposed [15–20]. While effective in limiting adhesion, these techniques typically require complex and time-consuming fabrication processes such as photolithography or dry etching, and the resulting substrates are difficult to reuse due to challenges in sterilization and cleaning, leading to high costs. Consequently, such surfaces are not suitable for the industrialization of cell-based applications. There is a need to develop low-adhesion cell culture surfaces that are easy to fabricate, reusable, and compatible with cell culture.

**Fig. 1:**
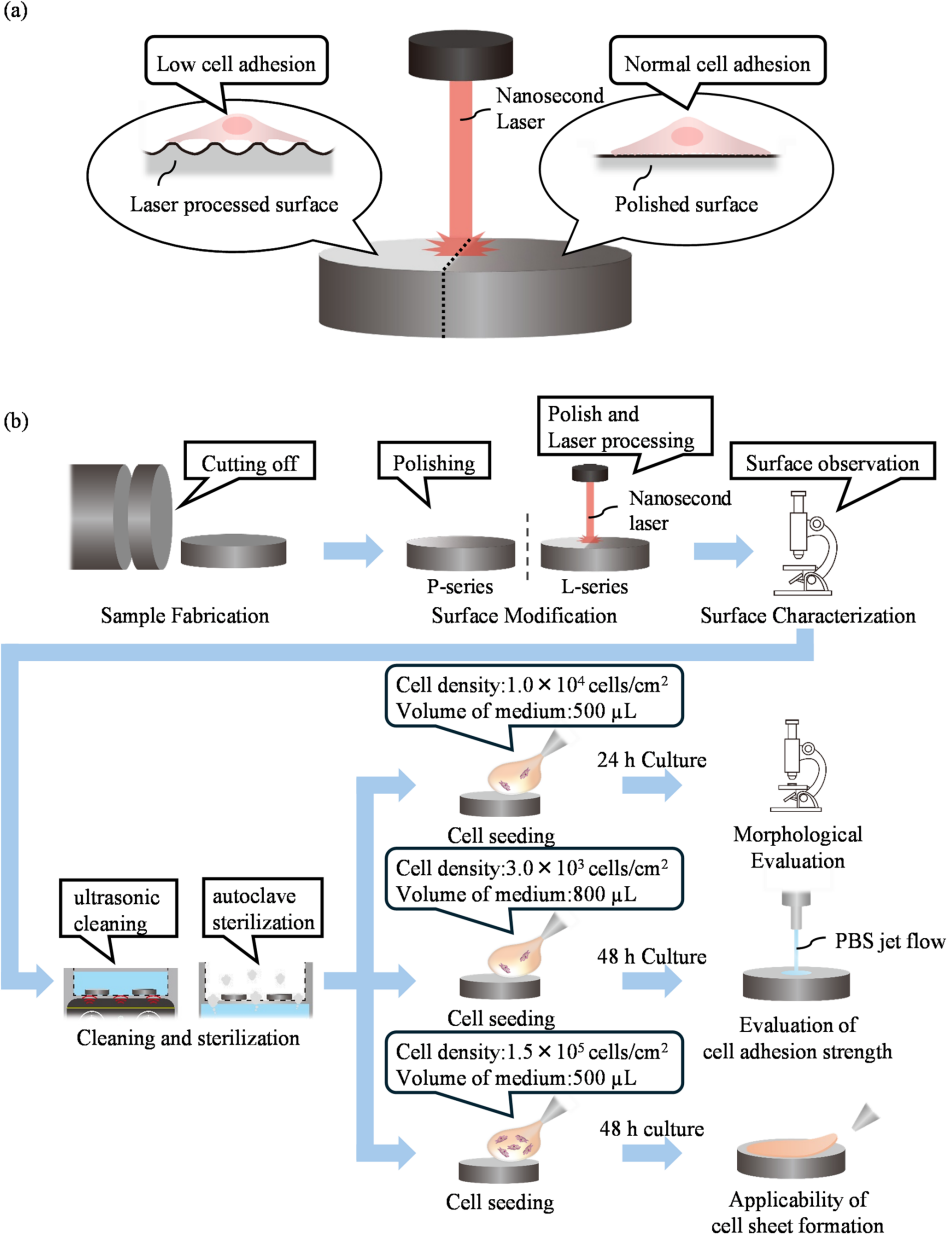
Schematic of the research concept and experimental workflow. **a** Concept of the fabrication process for a low-adhesion metallic cell culture surface using nanosecond laser processing. **b** Overview of the experimental workflow, including surface fabrication, cell seeding, incubation, and subsequent evaluation

In the present study, we developed a reusable low-adhesion cell culture surface by rapidly and simply fabricating microstructures on a metallic substrate using nanosecond pulsed laser processing (Fig. 1a). The cytocompatibility and reusability of the developed cell culture surface have already been confirmed [21]. Furthermore, previous studies have shown that metallic cell culture surfaces possess cytocompatibility and reusability [22–24]. By suppressing the adhesion area, the developed surface effectively reduced cell adhesion and enabled easy detachment of cells through the trigger of physical stimulation. We demonstrated both single-cell detachment for subculture purposes and the detachment of cell sheets for tissue engineering. This approach holds promise for applications in cell sheet engineering and enzyme-free cell collection technologies.

## Result

### Fabrication of low-adhesion metallic culture surface

**Fig. 2:**
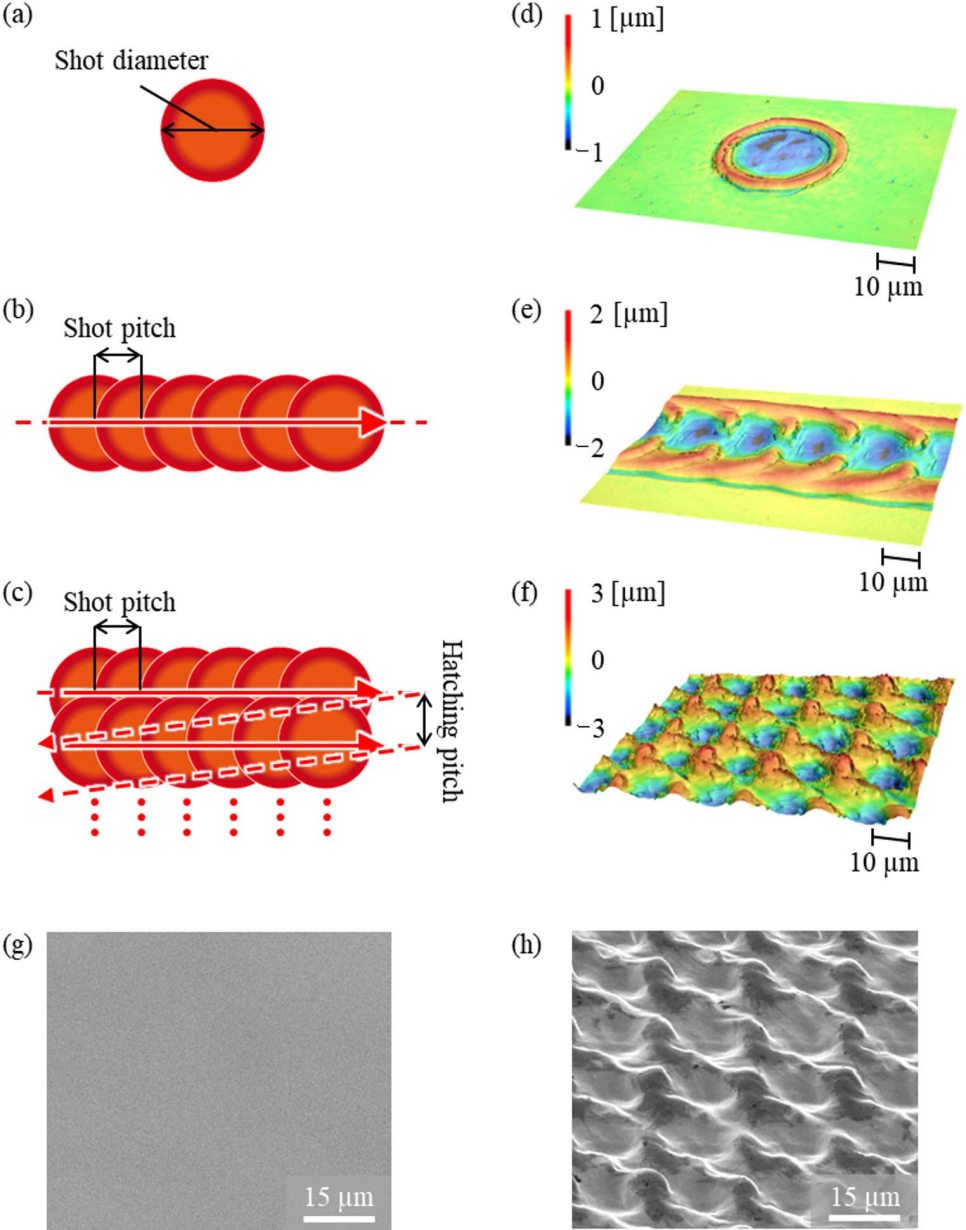
Laser processing microstructure fabrication method **a** concept of Single-shot laser processing, **b** line laser processing, **c** overlapping laser processing. **d** 3D surface height profile after single spot laser-processing, **e** after line laser-processing, **f** after overlapping laser-processing, SEM images of cell culture surfaces: **g** polished surface (P-series), **h** polished and laser-processed surface (L-series).Color bars in (**d**–**f**) indicate surface height (µm); note that the scales differ between panels

As a culture surface, a disk-shaped metallic sample (diameter: 20 mm, thickness: 3 mm) was prepared by cutting from a Ti-6Al-4 V alloy rod (Fig. 1b). Prior to laser processing, the surface of the sample was mirror-polished to ensure uniformity and minimize surface roughness. To develop micro- and nano-scale surface roughness smaller than the typical cell size (~ 30 μm) [25], nanosecond pulsed laser processing was employed to fabricate protrusions smaller than 10 μm and inter-protrusion spacing smaller than 15 μm. First, to determine the optimal laser parameters capable of creating fine surface structures, single-shot laser irradiation was conducted as shown in Fig. 2a. As a result, ring-shaped structures were formed under specific conditions (Fig. 2d). Among the developed rings, the smallest had a height of 2 μm, inner diameter of 20 μm, and outer diameter of 30 μm. It was considered that partially overlapping these rings would allow the development of fine structures at the desired scale.

Next, laser scanning intervals in both the horizontal and vertical directions were systematically varied to develop microstructures (Fig. 2b). In this laser processing method, the interval and shape of the resulting microstructures can be modulated by adjusting the shot pitch and hatching pitch. When the spacing was set to 20 μm, regularly aligned surface structures were formed (Fig. 2e). Then, laser processing was performed with a horizontal spacing of 20 μm and a row spacing of 15 μm (Fig. 2c), resulting in fine surface topography as shown in Fig. 2f. In this study, two types of culture surfaces were prepared: the P-series, consisting of a mirror-polished titanium surface (Fig. 2g), and the L-series, consisting of a mirror-polished and laser-processed titanium surface (Fig. 2h). The L-series exhibited regularly patterned protrusions with a 15 μm pitch, which is smaller than the typical cell size (~ 30 μm), indicating that microstructures of a scale smaller than cells were successfully developed. Details of the processing parameters (Supplementary Table 1) and fabricated surface structures (Supplementary Fig. 1.) are provided in the Supplementary Information.

### Evaluation of cytocompatibility of the developed culture surface

**Fig. 3:**
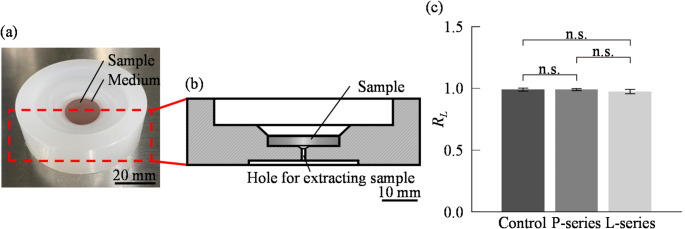
Development and evaluation of cell culture set up **a** Overview of cell culture setup, **b** cross-sectional view of cell culture setup, **c** ratios of live cells to all cells (*R*_*L*_) after 24 h culture. (*N* = 3; mean ± SD

To conduct cell culture experiments on the developed surfaces, a polypropylene culture chamber was fabricated and employed to hold the metallic culture surfaces, as shown in Figs. 3a and b. The fabricated culture surface was disk-shaped and was used by fitting it into a polypropylene culture chamber. After each experiment, the culture surface was retrieved by pushing it out from the backside using a thin rod through the hole for extracting sample. The chamber is autoclavable and, like the culture surface, can be reused by performing ultrasonic cleaning with neutral detergent (7X) and ultrapure water followed by autoclave sterilization. To evaluate cytocompatibility in this constructed culture environment, cells were seeded on polystyrene dishes, P-series, and L-series surfaces, and their viabilities after 24 h of incubation were evaluated. In this study, we used the mouse-derived myoblast cell line C2C12 as a representative adherent cell line, owing to its robust adhesion properties, and ease of handling, which make it suitable for initial validation experiments. As shown in Fig. 3c, the cell viability on the P-series and L-series surfaces was comparable to that on plastic dishes, which shows good cytocompatibility of the developed culture surface.

### Morphological evaluation of cell adhesion

**Fig. 4:**
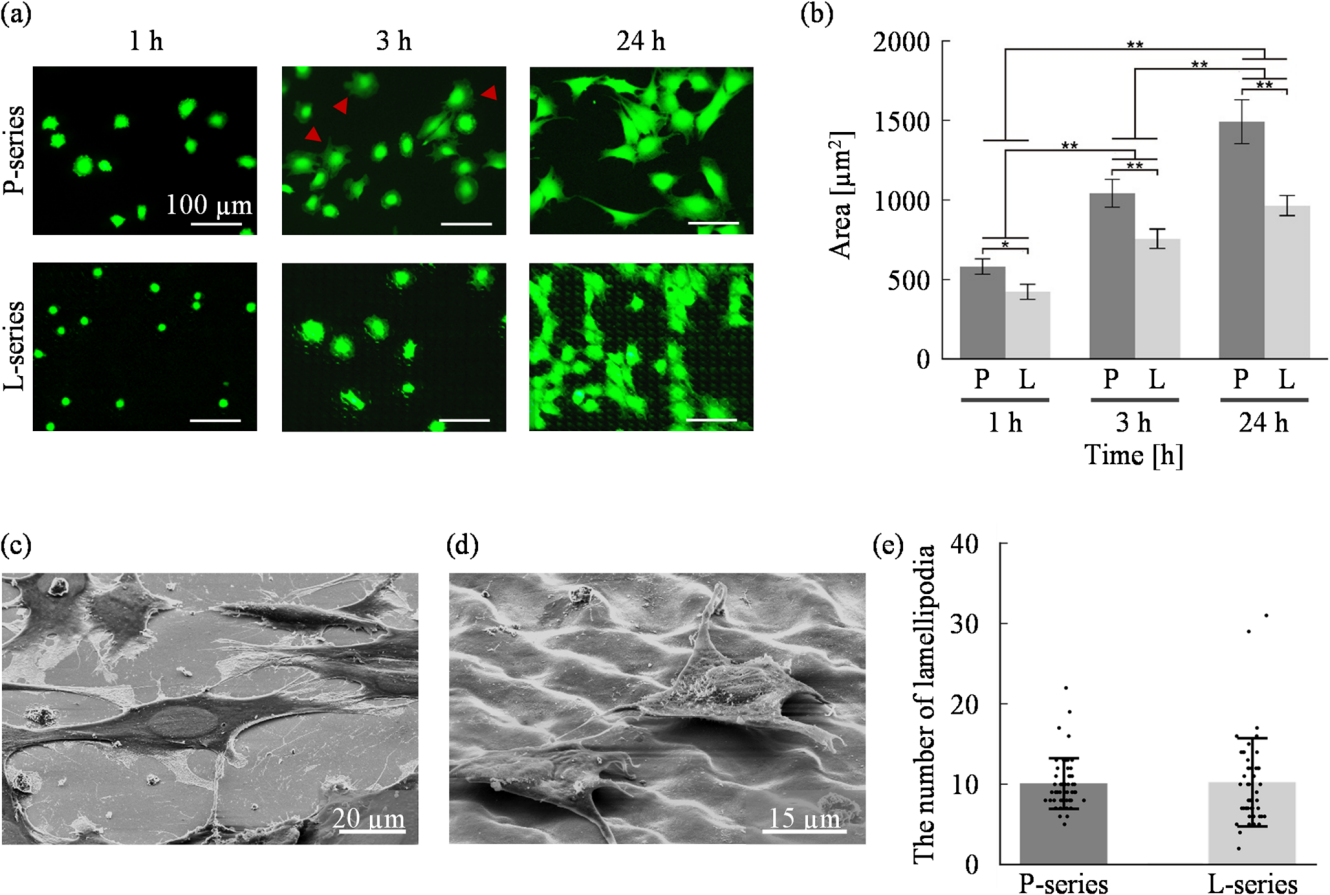
Cell adhesion evaluation based on cell morphology on engineered metallic cell culture surfaces. **a** Fluorescence time-lapse images showing adhesion and spreading of cells cultured on P- and L-series surfaces. Cells are stained with Calcein-AM. The red arrows show the extended lamellipodia, **b** Cell spreading area at different culture times (*N* = 3; mean ± SD; *p < 0.05, **p < 0.01) SEM images of cells cultured on different surfaces for 24 h (tilt angle: 50°) : **c** P-series and **d** L-series. **e** Number of lamellipodia of the cell cultured for 3 h (*N* = 3; mean ± SD)

As shown in Fig. 4, cells were cultured for 24 h on the developed surfaces, and their adhesion morphology was evaluated. To assess the effect of surface microtopography on cell adhesion morphology, P-series and L-series surfaces were used. In this experiment, cells were seeded on a circular culture surface with a diameter of 20 mm at a seeding density of 1.0 × 10⁴ cells/cm² with 500 µL of culture medium. To evaluate the cell adhesion of the developed culture surface, this study assessed cell adhesion based on qualitative and quantitative evaluation. We hence evaluate two-dimensional adhesion morphology by fluorescence staining images, three-dimensional morphology by scanning electron microscopy (SEM), and adhesion strength based on detachment behavior in response to physical stimuli induced by PBS jet flow and pipetting.

To quantify the effect of the microtopography on adhesion morphology, the average area of individual live cells was measured using fluorescence images obtained at each time point. As shown in Fig. 4b, a cell area increased significantly over time on both P- and L-series surfaces, and cell area on the L-series was significantly smaller than that on the P-series at each time point. Two-way ANOVA confirmed that these differences were statistically significant, indicating that the proposed surface inhibits the extension of lamellipodia.

To evaluate the effect of surface microtopography on cell adhesion in three dimensions, cells cultured for 24 h were observed by scanning electron microscope (SEM) following the method described by Inoue et al. (1988) [26]. As shown in Figs. 4c and d, cells on the L-series selectively adhered their lamellipodia to the protrusions of the microstructures, in contrast to the more broadly spread morphology on the P-series. To evaluate the effect on cell adhesion quantitatively, the number of lamellipodia per adherent cell on the culture surface was counted. Measurements were taken after 3 h of culture (Fig. 4e), because individual counting became difficult after 24 h due to cell–cell contact, whereas cell adhesion on the L-series was insufficient at 1 h. As a result, cells on the P-series exhibited a relatively small variation in lamellipodia counts, generally displaying a uniform adhesion pattern compared to the L-series.

### Evaluation of cell adhesion strength

**Fig. 5:**
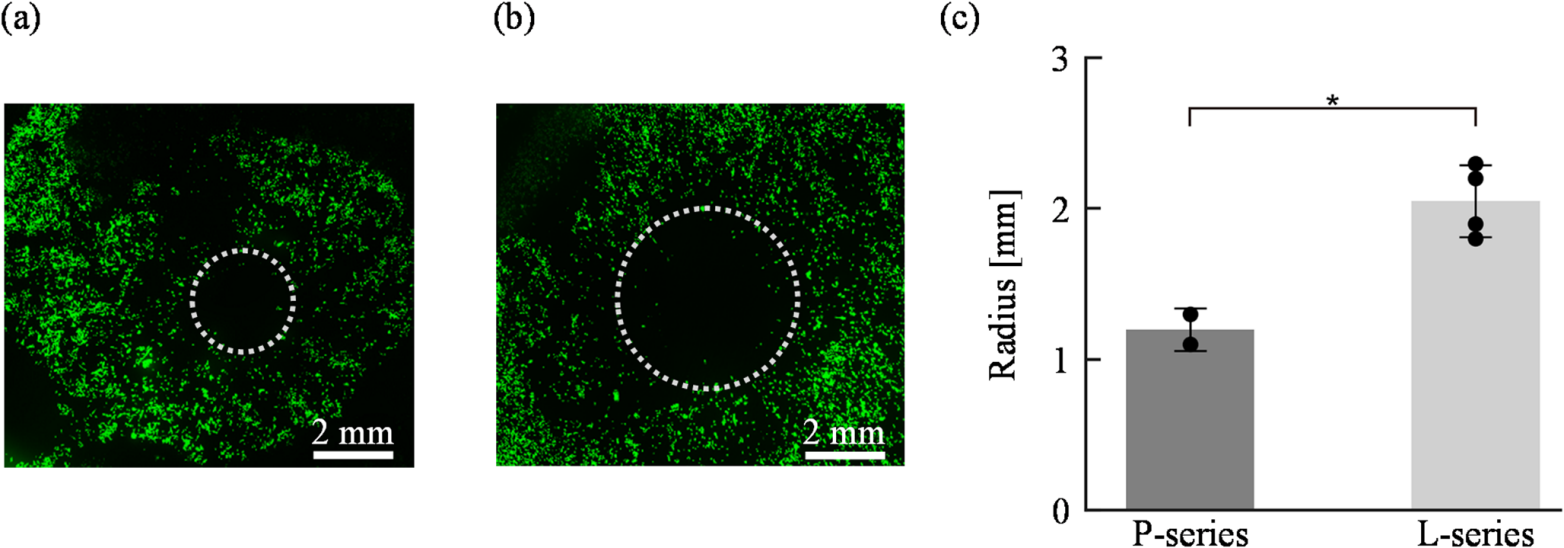
Evaluation of cell adhesion strength on engineered metallic culture surfaces using shear stress-induced detachment generated by PBS jet flow. Typical fluorescence images of detachment zones formed by shear stress on different culture surfaces: **a** P-series and **b** L-series. **c** Quantitative comparison of detachment zone radii on P-series and L-series surfaces. (mean ± SD; *p* < 0.05) The dotted circle indicating the cell detachment area was drawn in accordance with Supplementary Fig. 3

To compare adhesion strength between cells cultured on P-series and L-series surfaces, cell adhesion strength was evaluated using PBS jet flow. Cells were seeded on the culture surface at a density of 3.0 × 10³ cells/cm² with 800 µL of culture medium and cultured for 48 h. For this experiment, instead of using the culture chamber shown in Figs. 3a and b, a PDMS ring (outer diameter: 20 mm, inner diameter: 15 mm) was placed onto the culture surface to define the culture area (see the detail in Supplementary Fig. 2). Then, a constant flow of PBS (flow rate: 1.72 mL/s, duration: 3 s) was applied from a tubing pump (Front Lab Tubing Pump Set, Single Lever Down, 480 mL/min, FP300-1515) fixed at a constant height above the culture surface to ensure uniform application, detaching the cells from the surface. Figures. 5a and b show the results of evaluation of cell adhesion strength by PBS jet, and Fig. 5c shows the measured radii of detachment zones. As shown in Figs. 5a–c, cells on the L-series formed significantly larger detachment zones than those on the P-series. Since shear stress decreases with increasing distance from the impact point of the flow, this result indicates that the microstructured surface reduces adhesion strength. As described in the Supplementary Information (Supplementary Note 1), the shear stress required to detach cells on the L-series was estimated to be about 62% of that on the P-series. In other words, the adhesion strength on the L-series was suppressed to approximately 62% of the P-series value.

### Formation and detachment of cell sheets

**Fig. 6:**
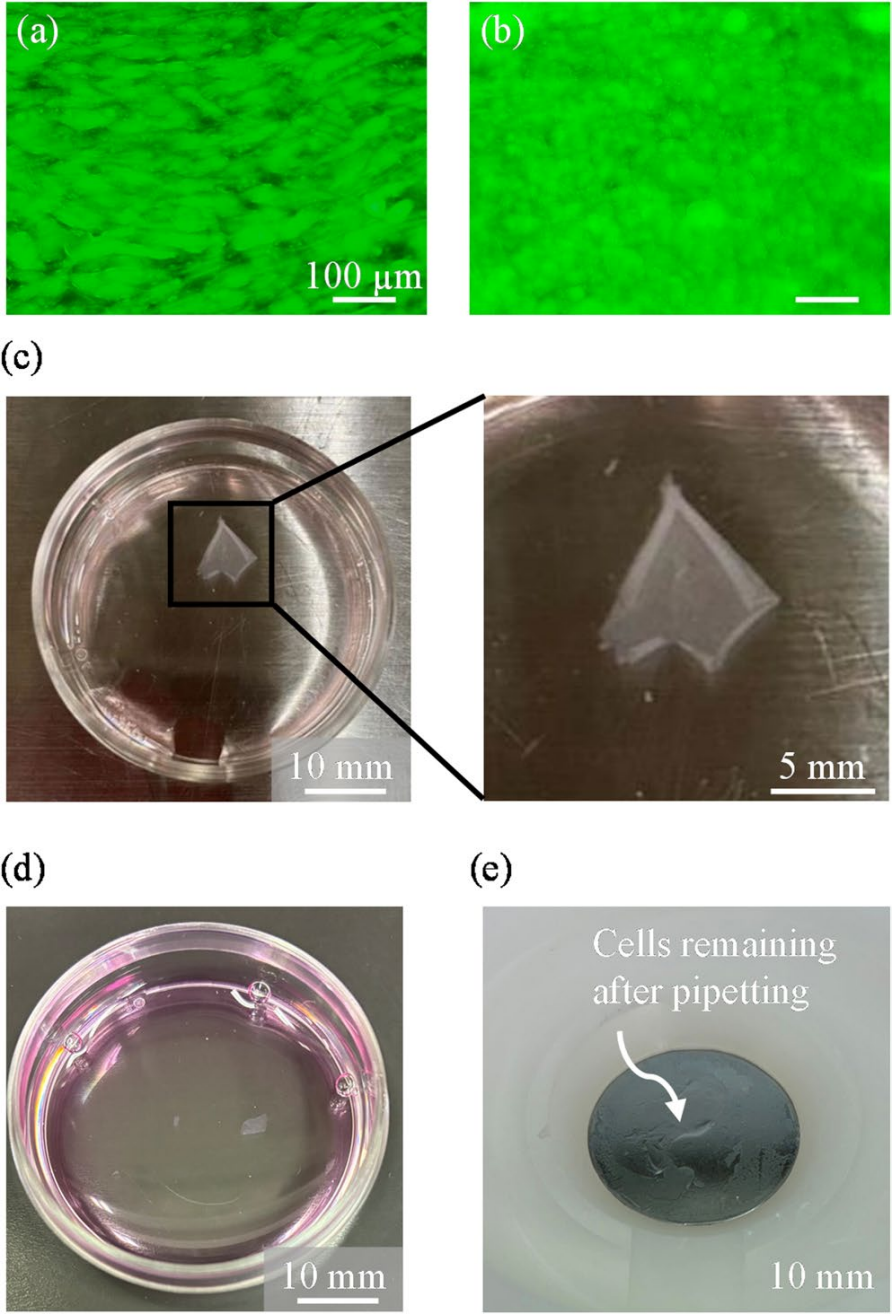
Evaluation of the applicability of the developed culture surface for cell sheet detachment. Fluorescence images of confluent cells before detachment: **a** P-series; **b** L-series. **c** Cell sheet detached from the L-series culture surface by pipetting. **d** Cells detached from the P-series by pipetting. **e** Cells remaining on the P-series culture surface after pipetting

To evaluate the applicability of the developed culture surface for tissue engineering, we examined whether cell sheets could be harvested using this surface. To generate over-confluent monolayers as shown in Figs. 6a and b, cells (seeding density of 1.5 × 10⁵ cells/cm² with 500 µL of culture medium) were seeded on both P-series and L-series surfaces and cultured for two days. Cell sheet detachment was then conducted by pipetting using a 200 µL micropipette. As shown in Fig. 6c, cells on the L-series were easily detached in sheet form by pipetting. The detached cell sheet collected from the L-series surface contracted to 19.6% of its original area prior to detachment, which is consistent with previous reports [9, 27]. In contrast, as shown in Fig. 6d, cells on the P-series could not be detached as sheets; instead only small cell clusters were recovered. Furthermore, some cells remained adhered to the culture surface as shown in Fig. 6e.

## Discussion

In this study, we aimed to develop a culture surface with low cell adhesion by a rapid and simple development method creating microscale surface structures on metallic culture surfaces using laser processing [28–31]. Under controlled conditions, reproducible roughened surfaces could be developed [32–36], indicating the high versatility of this method for experimental use. In addition, the metallic culture surface used in this study exhibited excellent chemical and mechanical stability and could be reused after autoclave sterilization, which is suitable for industrial applications in cell-based technologies from the viewpoint of the cost and sustainability [21–24].

In this study, the cell adhesion to the developed cell culture surface was comprehensively evaluated based on both morphological observations and physical detachment behavior. Fluorescence imaging revealed that cells cultured on the L-series cell culture surface exhibited suppressed lamellipodia formation, smaller spreading areas, and maintained a rounded morphology compared to those on the P-series, which indicates impaired adhesion based on previous studies (Fig. 4a). Furthermore, quantitative analysis demonstrated that the increase in cell area over time was delayed on the L-series, suggesting that not only initial adhesion but also the subsequent strengthening of adhesion was inhibited (Fig. 4b). These morphological findings strongly implied that the microstructured surface effectively suppresses the development of firm cell–culture surface interactions. To verify these implications, physical detachment tests were performed (Fig. 1b). In the evaluation of cell adhesion strength, cells on the L-series cell culture surface exhibited significantly larger detachment zones than those on the P-series under the same flow conditions, confirming a reduction in adhesion strength (Fig. 5). In addition, the evaluation of applicability of cell sheet formation showed that cells on the L-series could be detached as intact sheets, while cells on the P-series remained adherent (Fig. 6). This sheet-like detachment suggests that the developed cell culture surface weakens the cell–culture surface interface specifically, without disrupting cell–cell junctions or extracellular matrix (ECM) integrity. Together, these results demonstrate that the engineered microstructured cell culture surface not only alters the morphology of adherent cells but also effectively weakens their adhesion strength, enabling controlled and gentle detachment without enzymatic treatment, and suggesting its applicability in cell sheet formation technologies. These findings also indicate that, on the developed cell culture surface, cells can remain adherent under normal culture conditions but can be detached on demand when physical triggers such as fluid shear stress is applied. This controllable adhesion behavior demonstrates the potential of the developed culture surface for applications requiring precise timing of cell release.

The observed reduction in cell adhesion on the developed surface is considered to result from interference with physical contact between the cells and the culture surface. This effect arises from microstructures formed by laser processing under conditions designed, based on previous reports [37], to ensure that both the protrusions and their spacing were smaller than the typical cell size, specifically less than 15 μm. Direct contact between adhesion molecules and the culture surface is essential for cell adhesion, but the microtopography of cell culture surface reduced the contact area, thereby lowering adhesion [38, 39]. In addition, the discontinuity of the contact surface due to the microtopography likely hindered the formation of lamellipodia, preventing cells from spreading and resulting in a smaller adhesion area. Such topographical features may also affect cytoskeletal organization and intracellular tension, potentially delaying or suppressing the activation of adhesion-related signaling pathways. Accordingly, suppression of lamellipodia formation at the initial adhesion stage on laser-processed surfaces may affect the long-term stability of cell adhesion and cellular functions [39–41]. Because the present study focused on short-term behavior, further studies are warranted to elucidate these effects over extended culture. The presence of gaps between the cells and culture surface may have allowed the culture medium to infiltrate the interface, destabilizing adhesion and promoting detachment. Furthermore, cells cultured on the microstructured surface exhibited limited spreading, forming thicker vertical morphologies. This likely increased the effective surface area exposed to fluid shear stress, making the cells more susceptible to detachment by shear forces. Since we consider that the effects observed on the developed culture surface are primarily attributable to its surface topography, similar cell adhesion–suppressing characteristics may be observed in other types of adherent cells. However, systematic cross-cell-line validation will be necessary for broader generalization.

In this study, the developed cell culture surface demonstrated reduced cell adhesion due to the presence of laser-processed microstructures on its surface. This technology presented in our study can be combined with enzyme-free detachment techniques—such as temperature-responsive polymers and ultrasound—for detaching cells with minimal stress. Importantly, such enzyme-free approaches allow cells to be detached while preserving cell–cell junctions and adhesion-related extracellular proteins, including components of the extracellular matrix (ECM), thereby maintaining cellular functionality and viability for downstream applications in tissue engineering. Additionally, because the developed cell culture surface exhibits weak adhesion to cells, it allows for more gentle enzymatic detachment during subculturing. For instance, cells can be harvested with shortened trypsin exposure or using diluted trypsin solutions, thereby improving post-harvest cell viability. Reducing the physical and chemical stress during detachment is essential for maintaining cell quality in extended culture processes.

Furthermore, this study confirmed that cells could be detached from the developed culture surface in the form of intact sheets. This suggests that the surface selectively weakens the adhesion between the cells and the culture surface, while preserving cell–cell junctions and interactions with the extracellular matrix (ECM). As a result, cells can be harvested as structurally cohesive sheets, supporting their potential application in cell sheet engineering. In this study, manual pipetting imposed localized edge shear on the detached sheets, leading to peripheral edge damage. For industrial-scale production of cell sheets using the developed culture surface, it would be preferable to avoid manual operation and instead apply medium at a constant pressure and flow rate using devices such as syringe pumps. Furthermore, since the developed culture surface may influence the ability to form cell sheets, it will be necessary in future studies to examine cell–cell junction markers, which could affect the feasibility of sheet retrieval.

Notably, the nanosecond laser processing method used in this study allows precise control over the scale and spacing of the microstructures by adjusting the irradiation parameters. Because this technique can fabricate a wide variety of surface patterns, including SI type topographic patterns (Supplementary Fig. 1b-e), it will be important in future work to evaluate cell adhesion properties of these surfaces and systematically investigate the effects of periodicity, amplitude, and anisotropy. This enables tailored modulation of cell adhesion depending on the cell type or experimental purpose, providing high versatility and reproducibility for a wide range of cell-based applications. In future work, it will be important to evaluate whether cells can stably adhere and proliferate on the developed cell culture surface to ensure its suitability as a platform for both reliable and efficient cell expansion and on-demand detachment. Additionally, since previous studies [42] have reported that surface roughness reduces cell adhesion and enhances cell migratory behavior, analyzing the migration patterns and velocities of cells on the developed surface may provide further evidence supporting the reduced adhesion properties of the microstructured culture surface. Finally, practical implementation of this technology is feasible by fabricating metallic well plates or culture dishes and applying the same laser processing conditions to their flat bottom surfaces as used in this study. Since both formats feature planar bottoms, the established laser processing protocol can be directly applied without modification.

## Materials and methods

### Surface modification and characterization of culture surfaces

To develop the culture surface, nanosecond pulsed laser processing was employed. This technique utilizes the thermal energy of laser irradiation to melt and resolidify the surface of the metallic culture surface, thereby forming microscale surface structures smaller than the size of cells. Prior to laser processing, the metallic disk samples were mirror-polished to ensure consistent surface roughness. Polishing was performed using an automatic polishing machine (MECATECH250, PRESI) with waterproof abrasive papers of #120, #240, #400, #600, #800, and #1200 grits in sequence, followed by final polishing with a colloidal silica suspension. The detailed laser processing conditions are summarized in Supplementary Table 1.

After surface modification, a 3D surface profile was acquired using a laser scanning microscope (VK-X1000, KEYENCE). Additionally, the morphology of the developed microstructures was examined using an environmental scanning electron microscope (E-SEM).

### Cell culture

C2C12 murine myoblasts were cultured in Dulbecco’s modified Eagle’s medium (D-MEM, Thermo Fisher Scientific) supplemented with 10% fetal bovine serum (FBS, Funakoshi Co.) and 1% penicillin-streptomycin (Thermo Fisher Scientific) under 5% CO₂ at 37 °C. Cells were passaged using 0.05% trypsin-EDTA (Life Technologies). For maintenance, cells were cultured in standard plastic culture dishes.

To evaluate biocompatibility (Fig. 3c), cells were seeded at a density of 1.0 × 10⁴ cells/cm² on the metallic culture vessels and cultured for 24 h. For comparison, the same seeding density was used for culture in 35 mm plastic dishes. After incubation, viable and non-viable cells were stained with Calcein-AM and propidium iodide (PI), respectively. The percentage of the viable cell number was quantified to assess cell viability.

### Cell fixation for morphological analysis

To observe cell morphology, cells were first cultured for 24 h and then gently rinsed with PBS to avoid detachment. The cells were fixed in 2.5% glutaraldehyde in PBS at 4 °C for 30 min, followed by two washes with PBS. Stepwise dehydration was performed by immersing the samples sequentially in 20%, 50%, 80%, 90%, and 95% ethanol for 15 min each. Subsequently, the samples were washed once with 99.5% ethanol and then immersed in fresh 99.5% ethanol for 30 min to ensure complete dehydration. Ethanol was replaced with t-butyl alcohol, and the samples were incubated for 15 min before undergoing freeze-drying. An osmium coating was then applied to impart conductivity using an osmium coater (HPC-20) for 5 s, and the cell morphology was observed using a field-emission scanning electron microscope (FE-SEM) at an accelerating voltage of 5 kV, a working distance of 11.8 mm, and a tilt angle of 50°.

### Image analysis

Quantification of the cell adhesion area was performed by staining the cells with Calcein-AM after 24 h of culture. Fluorescence images were captured from five randomly selected regions per sample. Using ImageJ software (NIH, USA), both the total area of viable cells and the number of cells were automatically measured. The average adhesion area per cell was then calculated by dividing the total cell area by the cell count. The mean value obtained from the five regions was used as the representative value for each sample.

### Cleaning of culture vessels

The culture containers were cleaned by soaking them overnight in 7X cleaning solution (Funakoshi Co.), followed by ultrasonic cleaning for 15 min each in both the cleaning solution and ultrapure water. The cleaned containers were then dried, sealed in sterile packaging, and sterilized by autoclaving. They were opened and assembled in a clean bench immediately before the experiment.

### Statistical analysis

To evaluate the effects of culture time and culture surface structure on cell adhesion, two-way analysis of variance (two-way ANOVA) was performed with a significance level of α = 0.05.

## Supplementary Information

Below is the link to the electronic supplementary material.

Supplementary Material 1

## Data Availability

No datasets were generated or analysed during the current study.
