# Supplementary material for: Fabrication of a low-adhesion metallic cell culture surface by nanosecond laser processing
Source: Bioprocess Biosyst Eng. 2025 Dec 20;49(3):505–15. doi: 10.1007/s00449-025-03268-5 (PMC13021703; doi:10.1007/s00449-025-03268-5)
Supplement: Supplementary file 1 — Supplementary Material 1 [file 449_2025_3268_MOESM1_ESM.docx]

**Supplementary Materials**

**Fabrication of a Low-Adhesion Metallic Cell Culture Surface by Nanosecond Laser Processing**

**Kaisei Ito^1^, Atsushi Ezura^2^, Hideharu Shimozawa^3^, Yoshikatsu Akiyama^4^, Chikahiro Imashiro^5^, Jun Komotori^6^**

**Correspondence should be addressed to Chikahiro Imashiro, Jun Komotori**

**E-mail:[imashiro@pe.t.u-tokyo.ac.jp](mailto:imashiro@pe.t.u-tokyo.ac.jp)**, **[komotori@mech.keio.ac.jp](mailto:komotori@mech.keio.ac.jp)**

1. **School of Science for Open and Environmental Systems, Graduate School of Science and Technology, Keio University, Kanagawa 223-0061, Japan.**
2. **Sanjo City University, 5002-5 Kamisugoro, Sanjo-shi, Niigata 955-0091, Japan.**
3. **Medical Systems and Components Operations, Canon Inc., 3-30-2 Shimomaruko, Ota-ku, Tokyo 146-8501, Japan.**
4. **Institute of Advanced Biomedical Engineering and Science, Tokyo Women's Medical University (TWIns), 8-1 Kawada-cho, Shinjuku-ku, Tokyo 162-8886, Japan.**
5. **Graduate School of Engineering, The University of Tokyo, Hongo 7-3-1, Bunkyo-ku, Tokyo 113-8654, Japan.**
6. **Department of Mechanical Engineering, Keio University, Yokohama, Kanagawa 223-0061, Japan.**

**Supplementary Table.1. Laser processing parameters for surface microstructure fabrication**

| **Series name** | **A (L-series)** | **B** | **C** | **D** | **E** |
| --- | --- | --- | --- | --- | --- |
| **Output [W]** | **13.7** | **13.7** | **13.7** | **13.7** | **13.7** |
| **Repetition rate [kHz]** | **100** | **100** | **100** | **100** | **100** |
| **Pulse width [ns]** | **200** | **200** | **200** | **200** | **200** |
| **Scanning speed [m/s]** | **2** | **2** | **2** | **1.5** | **1** |
| **Shot pitch [µm]** | **20** | **20** | **20** | **15** | **10** |
| **Hatching pitch [µm]** | **15** | **20** | **10** | **15** | **10** |

**Supplementary Fig.1. 3D surface profiles of laser-processed surfaces under various irradiation conditions.** (a) Shot pitch: 20 µm, hatching pitch: 15 µm (L-series), (b) Shot pitch: 20 µm, hatching pitch: 20 µm, (c) Shot pitch: 20 µm, hatching pitch: 10 µm, (d) Shot pitch: 15 µm, hatching pitch: 15 µm, (e) Shot pitch: 10 µm, hatching pitch: 10 µm.

**Supplementary Fig. 2. PDMS ring: configuration and dimensions (dimensions in mm)**

**Supplementary Fig. 3. Binarized fluorescence images of jet-induced cell detachment with best-fit circle overlays. (a) P-series; (b) L-series.**

**Supplementary Note.1.**

The wall shear stress $\tau\left( r \right)$ was computed following Phares et al. (2000) for an axisymmetric normally impinging jet using the dimensionless profile $g_{2}\left( r/H \right)$:

$$\tau\left( r \right)=\rho u_{0}^{2}Re_{0}^{-1/2}\left( \frac{H}{D} \right)^{-2}g_{2}\left( \frac{r}{H} \right) .$$

where $\rho, \nu, Q, D, H, u_{0}$and $Re_{0}$, and are the fluid density $\rho=993 kg/m^{3} \left( \mathrm{PBS} \right)$ and viscosity $\nu=0.90\times{10}^{-6} m^{2}/s$, volumetric flow rate $Q=1.72 mL/s$, nozzle diameter $D=7.0 \mathrm{mm}$, nozzle–plate distance $H=17 \mathrm{mm}$, nozzle-exit velocity$u_{0}=4Q/\left( \pi D^{2} \right)\approx4.47 m/s$, Reynolds number $Re_{0}=u_{0}D/\nu\approx3.48\times{10}^{3}$ (Phares et al. 2000. https://doi.org/10.1017/S002211200000121X). The function $g_{2}\left( r/H \right)$ as obtained from the universal curve reported by Phares et al. via numerical reading with spline interpolation.

Because $\rho, \nu, Q, D,$and $H$ were held constant, $\tau$ is effectively set by the detachment radius $r$ through $g_{2}(r/H)$; in the inner impingement region, $\tau$ increases approximately linearly with $r$. Accordingly, the shear stresses required for detachment on two surfaces can be compared from their detachment radii:

$$\frac{\tau_{L}}{\tau_{P}}=\frac{r_{P}}{r_{L}}=\frac{1.26 \mathrm{mm}}{2.03 mm}\approx0.621$$

which indicates that the minimum shear required to detach cells on the L-series was about 62% of that on the P-series, as determined from the detachment radii in Fig. 5c. In other words, the cell adhesion strength on the L-series was probably suppressed to approximately 62% of the P-series value.
